# Psychometric evaluation and adaptation of the COMICE questionnaire for Swedish midwifery students

**DOI:** 10.18332/ejm/196137

**Published:** 2024-12-19

**Authors:** Ingegerd Hildingsson, Lena Bäck, Anette Björk, Lisbeth Kristiansen

**Affiliations:** 1 Department of Health Science, Mid Sweden University, Sundsvall, Sweden; 2 Department of Women's and Children's Health, Faculty of Medicine, Uppsala University, Uppsala, Sweden

**Keywords:** basic skills, confidence, education, midwifery, students

## Abstract

**INTRODUCTION:**

The world needs skilled, well educated, and confident midwives, but there is a lack of instruments to measure confidence in the Swedish context. The aim was to psychometrically test and adapt the COMICE (Confidence Of Midwifery students on selected midwifery Competencies at completion of Education) questionnaire on Swedish midwifery students.

**METHODS:**

This is a national cross-sectional study of midwifery students from all 13 universities in Sweden in 2016–2017. The questionnaire comprised antenatal, intrapartum, postpartum and newborn care and underwent a principal component analysis. Analysis of variance was used to study associations with students’ background characteristics.

**RESULTS:**

A total of 238 students (78% response rate) completed the questionnaire. For antenatal care, two components were identified: performing routine midwifery assessments during pregnancy and identifying fetal and maternal risk factors while educating parents (KMO=0.895, p<0.001; Cronbach’s alpha=0.848 and 0.827, respectively). For intrapartum care, three components emerged: managing physiological birth and providing support, managing complicated labor and birth, and identifying and providing lifesaving measures (KMO=0.905, p<0.001; Cronbach’s alpha=0.917, 0.809, and 0.777, respectively). Postnatal and newborn care included managing uncomplicated care while educating parents and managing complications (KMO=0.941, p<0.001; Cronbach’s alpha=0.993 and 0.558, respectively). Younger students (<31 years) reported greater confidence in routine antenatal assessments compared to older students (mean: 27.43 vs 26.01, p=0.004), managing physiological births (mean: 65.41 vs 62.85, p=0.01), and handling complicated labors (mean: 20.37 vs 19.05, p=0.046).

**CONCLUSIONS:**

This study supports the Swedish version of the COMICE questionnaire as a tool to measure confidence among midwifery students in a Swedish context.

## INTRODUCTION

Fostering confident midwives is a responsibility for the higher education institutions (HEI) in collaboration with clinical placements, such as antenatal clinics, labor wards and postnatal wards. There are a variety of ways to become a midwife, but the length, requirements and content of midwifery education differs between countries^1^. The three pathways to become a midwife are post-nursing, direct entry and double degree (nurse and midwife)^1^. A global access to midwifery care could prevent several maternal and newborn deaths^2,3^. There is, however, a lack of midwives around the world^4^, and Sweden is no exception^5^.

### Long tradition of midwifery education in Sweden

Swedish midwifery education has a long tradition with more than 300 years of educated midwives. Historically, the Swedish midwife worked in women’s homes by assisting during labor and birth. Antenatal care was introduced during the 1930s and was fully developed at the same time that the majority of births were moved to hospitals. From having a good amount of continuity from a midwife caregiver, the care became fragmented. Swedish midwives usually work either at antenatal outpatient clinics or at hospital-based labor and postnatal wards. The field of midwifery currently covers all aspects of women’s sexual and reproductive health^6^.

Swedish midwifery education is a post-nursing education. After completing three years of nursing education at the university level with a Bachelor’s degree, an additional 18 months of midwifery education is required. Therefore, Swedish midwives have double authorizations in nursing and in midwifery. The midwifery education also includes a one-year Master’s degree in reproductive health. All Swedish midwifery students are required to assist at least 50 births before graduation^6^.

### Swedish Association of Midwives, member of ICM

The Swedish Association of Midwives, founded in 1886, is a member of the International Confederation of Midwives (ICM), a non-governmental organization that has existed for over 100 years and currently includes 136 midwifery organizations from 117 countries. ICM defines a midwife as follows: ‘A midwife is a person who has successfully completed a midwifery education program based on the ICM Essential Competencies for Midwifery Practice and the framework of the ICM Global Standards for Midwifery Education, recognized in the country where it is located; who has acquired the requisite qualifications to be registered and/or legally licensed to practice midwifery and use the title “midwife”, and who demonstrates competency in the scope of practice of the midwife’. The basic competencies, first established in 2002 and thereafter updated several times, suggest that antenatal care includes assessing the health status of women and fetal wellbeing; monitoring pregnancy; and promoting healthy behavior and guidance. For intrapartum care, the competencies cover skills to promote normal, physiological and safe labor and birth and prevent complications; provide care for the newborn; and initiate breastfeeding. Postnatal and newborn care also require skills in detecting and treating complications in women and newborns^7-9^.

### COMICE reflects competencies of midwifery skills

Based on the basic competencies of midwifery skills developed by ICM, an international project was initiated in 2015. The project was named COMICE (Confidence Of Midwifery students on selected midwifery Competencies at completion of Education: a multi-centric study); a questionnaire was developed in an Indian province^10-12^ and thereafter tested in seven African countries (Tanzania, Kenya, Uganda, Zimbabwe, Zambia, Malawi, and Somaliland)^13-15^. The questionnaire comprises the areas of antenatal, intrapartum and postpartum/newborn care.

*Rational*

In most countries, the extensive questionnaire underwent a principal component analysis (PCA) to investigate underlying factors or components and find relevant dimensions. However, this analysis was not previously performed in Sweden. It is highly recommended that instruments are tested and validated when used in a new population^16^. The aim was to psychometrically test and adapt the COMICE questionnaire in a Swedish context and identify the underlying components of the instrument.

## METHODS

### Study design and setting

A cross-sectional national study with an adaptation and psychometric evaluation component where students enrolled in all Swedish midwifery programs (n=13) were invited to participate. All 13 midwifery programs in Sweden participated in the study. The HEIs providing midwifery education were situated in universities belonging to a medical faculty (n=7) or at universities without a medical faculty (n=6). Data collection took place from January 2016 to January 2017 to coincide with the semester endings (January and June).

### Participants

A convenience sample of all midwifery students in Sweden, in their final semester of their education in 2016–2017 who were present during the time of data collection were eligible and invited to participate.

### Data sources and measurement

After receiving information about the study through the Swedish Midwives Association’s Educational Board, a formal request was sent out to the program directors at the universities, who accepted to participate in the recruitment and negotiated about the best period for data collection. One of the authors (LB) travelled to all the universities and was available in the classrooms for clarification and questions about the content of the questionnaire when the students were filling it in. It took approximately one hour to complete the questionnaire. The COMICE questionnaire was used to investigate the Swedish midwifery students’ confidence prior to their final exam. The questionnaire measured midwifery students’ self-assessment of confidence against four selected areas of ICM competencies: antenatal (22 skills), intrapartum (48 skills), postpartum (16 skills) and newborn (19 skills) care.

The origin of the questionnaire comes from an Indian study by Sharma et al.^10^. It was initially translated from the ICM skills statements in English to Gujarati, then reviewed by a group of midwifery teachers who rated the skill statements. The questionnaire was subsequently pilot tested with a group of students^10^.

### Adaptation of the COMICE questionnaire

To translate and transform the COMICE from English to the Swedish language and cultural context, we were inspired by the back-translation methodology of Brislin^17^. Accordingly, several principal steps were taken to secure a proper, idiomatically correct and functionally equivalent Swedish version of the COMICE (Table 1). After obtaining permission to use and translate the questionnaire into Swedish, the transformation was agreed upon after discussing wording and concepts in relation to education and language understanding. Thereafter, a blinded back-translation from Swedish to English was performed. Finally, comparisons were made between the two versions and thereafter pilot tested.

**Table 1:** Actions taken, actors, and changes induced in the stepwise procedure of translating and adjusting COMICE into a working Swedish version

*Action taken*	*Done by*	*Change*
1. Asking and obtaining permission to use and translate (COMICE) into Swedish language	A professor in midwifery and nursing science	Not adequate
2. Translation from English to Swedish	A bilingual researcher who is also a midwife and familiar with the content of the ICM	Not adequate
3. Adjusting and agreeing upon the transformation into a cross-cultural fit. Here, idiomatic expressions and concepts were discussed to reach the best understanding in a Swedish language and educational context.	This was done by a group of three senior midwives, two new midwives and one obstetrician in several meetings.	Some items seemed inappropriate for the Swedish context and were removed from the questionnaire. These skills mainly covered basic hygiene routines, basic nursing skills, and religion. Consequently, 21 items were removed from the original questionnaire, as all midwifery students in Sweden are registered nurses prior to midwifery education.
4. Blinded back-translation from the Swedish COMICE into English	Two bilingual researchers from Mid Sweden University, to whom the original COMICE wording was unknown	Not adequate
5. Comparison between the two versions of the original in the source language. One from the original (step one) and back-translated version (step four).	The investigating research team on the final version of the Swedish COMICE	Almost full agreement between the versions. Discussion and decision to pursue.
6. The Swedish version of the COMICE was piloted, and no revisions were needed.	28 students in one midwifery Master’s programme in Sweden answered the questionnaire.	All items made sense to the responding students; therefore, no revisions were needed.

In addition to the ICM competencies displayed in the COMICE in relation to confidence, some background information was collected. These questions covered the students’ age, sex, whether they had children, years of experience as a nurse and whether their midwifery school was connected to a medical university.

### Ethics

The study was approved by the university ethics committee (Ref. 2015/1850). The respondents were informed that only the researchers had access to the data, and that the findings would be published without identifiable information on the participants. The students were free to refuse participation and could leave at any time without further explanation.

### Analysis

Descriptive statistics were used to present the students’ background information (frequencies and percentages). The large data set was subjected to several principal component analyses (PCAs) for each of the areas of antenatal, intrapartum, and postpartum and newborn care, to reduce the number of items and gather information about the interrelationships among the variables^18^.

The Kaiser-Meyer-Olkin (KMO) value^19^ was first checked to assess the factorability of the data sets. The KMO value should be >0.6 for sufficient factorability, and Bartlett’s test of sphericity should be statistically significant (p<0.05)^20^.

The number of components to retain for each of the areas was guided by Kaiser’s criterion (eigenvalue of 1) and Cattell’s scree test by inspecting the scree plots. Further, Horn’s parallel analysis^21^ was used to compare eigenvalues in each of the areas (antenatal, intrapartum, postpartum/newborn care) in the present sample with randomly generated data sets of the same size, where only those eigenvalues exceeding the corresponding values from the data sets were retained. All items in the questionnaire with an eigenvalue of 1 and a loading >0.40 were included. Cronbach alpha coefficients were thereafter calculated for each component to assess reliability. All items in each component were thereafter summed to form subscales. The subscales in all areas were analyzed in relation to students’ background characteristics through analysis of variance^18^.

## RESULTS

A total of 349 students were enrolled in the last semester of their midwifery education in Sweden 2016–2017. Of these, 303 were present during data collection (87%). Of the 303 students that were present, 238 completed the questionnaire and were included in the analysis. They represent 68% of all midwifery students in the final semester and 78% of those present at the time of data collection. Internal missing values were 1–5 for the items in antenatal care, 1–6 in intrapartum care and 1–7 for postnatal/newborn care.

The mean age of the students was 31 years (range: 24–54 years), and they were all female. The majority (82%) was living with a partner and 63% had children. Just over half of the participants (53%) had worked as a nurse for >4 years before entering the midwifery education. The majority (58%) were enrolled at a university with a medical faculty.

### Principal component analyses

*Antenatal care*

Different numbers of components were investigated in each of the areas until the best solution was found. The KMO value of antenatal care was 0.895 and Bartlett’s test of sphericity <0.001, suggesting good factorability. The initial rotated solution comprised three components. Horn’s parallel analysis, however, only confirmed the solution of retaining two components. The two components’ solution explained 40.08% of the variance. The first component covered nine items and was labelled: ‘Performing routine midwifery assessments during pregnancy’. The second component included eight items and was called: ‘Identifying fetal and maternal risk factors and educating parents’. The first component included common antenatal health check-ups, such as measuring the fundal height and listen to fetal heartbeat, while the second component included identifying medical and obstetrical health risks. Cronbach alpha coefficients calculated for each domain were 0.848, and 0.827, respectively. An overview of the included items, the pattern and structure matrix are shown in Table 2.

**Table 2:** Result of the principal component analysis for antenatal care from a national cross-sectional study performed in 2016–2017 on midwifery students in Sweden (N=238)

*Items*	*Pattern matrix*		*Structure matrix*	
	*Component 1*	*Component 2*	*Component 1*	*Component 2*
Calculate the expected date for birth	**0.832**	-0.155	0.746	0.306
Listen to fetal heart rate	**0.790**	0.007	0.794	0.444
Document findings for each visit in appropriate register	**0.712**	-0.133		
Provide guidance and basic preparation for labor, birth and newborn care	**0.604**	0.040	0.626	0.374
Understand meaning of findings of fetal heart rate assessment	**0.595**	0.202	0.708	0.532
Take initial and ongoing antenatal history	**0.589**	0.152	0.673	0.478
Perform complete abdominal assessment; measure fundal height, lie position and presentation	**0.500**	0.143	0.579	0.420
Teach and/or demonstrate to mother how to decrease common discomforts during pregnancy	**0.479**	0.360	0.678	0.625
Explain findings of physical examination to mothers	**0.443**	0.282	0.599	0.527
Identify pregnancy complications, e.g. pre-eclampsia	-0.081	**0.795**	0.440	0.796
Take first-line management of medical and pregnancy complications based on evidence-bases national/local guidelines before referral for high level intervention	-0.125	**0.741**	0.285	0.672
Identify medical complication during pregnancy, e.g. diabetes and anemia	0.097	**0.697**	0.483	0.751
Give appropriate advice on nutritional requirements of pregnancy and the relationship with fetal growth	0.017	**0.665**	0.386	0.675
Assess maternal nutrition	-0.014	**0.647**	0.344	0.639
Provide health education to adolescents, women and families about normal pregnancy progress, danger signs and symptoms and when/how to contact midwife	0.334	**0.502**	0.612	0.687
To educate mothers and family for birth preparation	0.389	**0.454**	0.640	0.669
Assess fetal growth by manual measurements	0.066	**0.425**	0.301	0.461
**Cronbach alpha**	**0.848**	**0.827**		

Bold values indicate major loadings after rotation. Pattern matrix shows the unique contribution of each variable to components. Structure matrix shows the overall correlation of each variable with components. Components are the dimensions derived from the data that explain variance.

*Intrapartum care*

The KMO value for intrapartum care was 0.905 and Bartlett’s test <0.001. Three components were identified, and the KMO test indicated that the assumed samples were adequate and had good factorability. Horn’s parallel analysis confirmed the solution of retaining three components. This solution explained 42.21% of the variance. Cronbach alpha coefficients calculated for each domain were 0.917, 0.809 and 0.777, respectively. Table 3 presents the pattern matrix and structure matrix of the PCA for intrapartum care. The three components in intrapartum care were labelled: 1) ‘Managing physiological birth and providing support’, 2) ‘Managing complicated labor and birth’, and 3) ‘Identifying and providing lifesaving measures’. The first component comprised 19 items related to common midwifery tasks during an uncomplicated labor and birth, such as measuring uterine contractions, supporting women and assessing the progress of labor. The second component with 10 items involved hands-on manoeuvres during complicated labor and birth, as well as birth interventions such as performing an episiotomy. The third component with three items were about treatment of life-threatening conditions.

**Table 3:** Result of the principal component analysis for intrapartum care from a national cross-sectional study performed in 2016–2017 on midwifery students in Sweden (N=238)

*Items*	*Pattern matrix*			*Structure matrix*		
	*Component 1*	*Component 2*	*Component 3*	*Component 1*	*Component 2*	*Component 3*
Monitor progress of labor using partograph or similar tool for recording	**0.712**	-0.037	-0.054	0.687	0.233	0.033
Prepare for birth (equipment, labor room, etc.)	**0.680**	-0.013	0.049	0.672	0.247	0.161
Ensure adequate hydration, nutrition and non-pharmacological comfort measures in labor/birth	**0.677**	-0.073	0.109	0.659	0.213	0.212
Inspect placenta and membranes for completeness	**0.672**	0.112	-0.059	0.702	0.353	0.066
Take specific history and maternal vital signs in labor	**0.662**	-0.049	0.005	0.641	0.202	0.122
Provide opportunity for women to express their needs, choices during labor	**0.659**	-0.118	0.127	0.632	0.186	0.203
Provide physical and psychological support for woman and family, and promote normal birth	**0.653**	0.005	-0.038	0.641	0.235	0.084
Provide for bladder care including performance of urinary catheterization when indicated	**0.643**	-0.137	0.142	0.611	0.161	0.226
Document diagnosis and care in appropriate register	**0.638**	0.000	0.111	0.672	0.247	0.179
Clamp and cut the cord	**0.631**	-0.126	0.053	0.598	0.171	0.097
Perform fundal massage to stimulate postpartum uterine contraction and uterine tone	**0.610**	0.159	-0.025	0.673	0.407	0.106
Perform complete/accurate pelvic examination for dilatation, descent, presenting part, position, membranes status and pelvis for vaginal birth	**0.609**	0.647	0.143	0.647	0.363	-0.047
Help presence of support person in labor and birth	**0.602**	-0.060	-0.069	0.567	0.162	0.034
Identify abnormal labor patterns and initiate appropriate and timely intervention and/or referral	**0.591**	0.277	0.041	0.684	0.462	0.193
Stimulate or augment uterine contractility, using non-pharmacological agents/measures	**0.581**	-0.053	-0.002	0.603	0.373	0.071
Calculate time of uterine contractions	**0.572**	0.174	-0.005	0.650	0.427	0.115
Provide a safe environment for mother and infant to promote attachment (bonding)	**0.558**	0.000	-0.006	0.561	0.237	0.057
Stimulate or augment uterine contractility, using non-pharmacological agents/measures	**0.554**	0.144	-0.025	0.557	0.176	0.080
Perform appropriate hand manoeuvres for a vertex birth	**0.519**	0.038	0.127	0.573	0.341	0.141
Repair 1st and 2nd degree vaginal tears or episiotomy	0.266	**0.708**	-0.186	0.450	0.730	-0.036
Perform appropriate hand manoeuvres for face and breech deliveries	-0.091	**0.604**	0.143	0.173	0.596	0.247
Inspect the vagina and cervix for lacerations	0.326	**0.566**	-0.069	0.524	0.664	0.069
Perform an episiotomy	-0.027	**0.546**	0.020	0.212	0.545	0.149
Perform manual removal of placenta	-0.082	**0.543**	0.023	0.166	0.525	0.144
Identify cervical lacerations and provide first level care	-0.114	**0.496**	0.259	0.118	0.499	0.307
Management of prolapsed cord while requesting medical attention and/or awaiting transfer	0.008	**0.450**	0.387	0.212	0.537	0.456
Manage shoulder dystocia	-0.007	**0.441**	0.424	0.224	0.530	0.486
Manage cord around baby’s neck at birth	0.310	**0.440**	0.130	0.498	0.588	0.254
Manage fetal distress	0.366	**0.421**	0.193	0.561	0.608	0.316
Identify shock	0.127	-0.056	**0.836**	0.268	0.265	0.857
Initiate management of shock (intravenous, oxygen, warmth, position)	0.167	-0.045	**0.813**	0.312	0.279	0.842
Perform aortic compression	0.010	0.095	**0.546**	0.172	0.339	0.597
Estimate and record maternal blood loss	0.369	0.340	0.029	0.519	0.480	0.155
Administer local anesthetic to perineum for episiotomy or perineal repair	0.398	0.406	-0.192	0.503	0.476	-0.064
Manage postpartum bleeding and hemorrhage, using appropriate techniques and uterotonic agents	0.351	0.384	0.189	0.545	0.560	0.326
Assess effectiveness of uterine contractions	0.358	0.380	-0.142	0.491	0.488	0.018
Conduct active management of the 3rd stage of labor; administer uterotonic drug within a minute of birth of infant	0.077	0.132	0.140	0.158	0.208	0.165
**Cronbach alpha**	**0.917**	**0.809**	**0.777**			

Bold values indicate major loadings after rotation. Pattern matrix shows the unique contribution of each variable to components. Structure matrix shows the overall correlation of each variable with components. Components are the dimensions derived from the data that explain variance.

*Postnatal and newborn care*

The same procedure was performed for postnatal and newborn care. In the first attempt to perform a PCA, items related to only postnatal care were included in the model. It was suggested to retain two components, where one component only comprised one item (support to bereaved families). We also tried a forced one-component solution, but then this item did not load at all. Therefore, the items in postnatal and newborn care were collapsed, and the analysis revealed a solution of two components. The KMO value was 0.941 and Bartlett’s test of sphericity p<0.001, suggesting good factorability. A total of 50.88% of the variance was explained by the model. The two-component solution was confirmed by the parallel analysis. The two components were labelled: 1) ‘Managing uncomplicated postpartum and newborn care and educating parents’, and ‘Managing complicated postpartum and newborn care’. Cronbach alpha values for the two components were 0.883 and 0.858 (Table 4).

**Table 4:** Result of the principal component analysis for postnatal and newborn care from a national cross-sectional study performed in 2016–2017 on midwifery students in Sweden (N=238)

*Items*	*Pattern matrix*		*Structure matrix*	
	*Component 1*	*Component 2*	*Component 1*	*Component 2*
Document newborn diagnosis and care in registers	**0.849**	-0.104	0.784	0.427
Take selective history, including details of pregnancy, labor and birth	**0.809**	-0.163	0.706	0.342
Educate mother on care of self and infant after childbirth including signs/symptoms of possible complications	**0.787**	-0.049	0.757	0.484
Educate the family and the mother on importance of maintaining hygiene and recognizing signs of infections	**0.766**	0.010	0.772	0.489
Promote and maintain normal newborn body temperature through covering (blanket, cap), environmental control and promotion of skin-to-skin contact	**0.753**	-0.107	0.687	0.365
Palpation of uterus, bleeding control, blood pressure	**0.740**	0.014	0.749	0.478
Position infant to initiate breastfeeding as soon as possible after birth and support exclusive breastfeeding	**0.735**	0.039	0.759	0.499
Perform a screening/physical examination of newborn for congenital defects	**0.691**	-0.054	0.658	0.378
Assess immediate condition of newborn (e.g. Apgar scoring)	**0.687**	0.159	0.786	0.588
Educate the family and the mother for appropriate postnatal rest and exercises	**0.681**	0.067	0.722	0.493
Educate parents about normal growth and development of the infant and young child, and how to provide for day-to-day needs of the normal child	**0.663**	0.228	0.806	0.643
Document postnatal care and complications in appropriate registers	**0.659**	0.092	0.717	0.505
Educate a woman and her partner on resuming sexual activity following childbirth	**0.618**	0.014	0.627	0.401
Provide family planning counselling and services as part of postpartum care	**0.603**	0.115	0.674	0.492
Initiate and support immediate and exclusive breastfeeding	**0.579**	0.072	0.624	0.435
Provide routine newborn care, as per local guidelines and protocols (e.g. identification eye care, screening tests, administration of vitamin K, birth registration)	**0.503**	0.283	0.680	0.597
Assist parents to access community resources	**0.474**	0.234	0.620	0.530
Assess for healing of lacerations and/or repairs	**0.470**	0.136	0.555	0.430
Identify complications for low birth weight and refer	0.033	**0.802**	0.535	0.823
Begin emergency measures for hypothermia	-0.030	**0.775**	0.455	0.756
Transfer the at-risk newborn to emergency care facility	-0.028	**0.773**	0.456	0.756
Give appropriate care to the low-birth-weight baby including kangaroo mother care	-0.009	**0.753**	0.462	0.748
Begin emergency measures for hypoglycemia	0.085	**0.740**	0.548	0.793
Begin emergency measures for respiratory distress (newborn resuscitation)	0.034	**0.734**	0.493	0.755
Support and educate parents who have given birth to multiple babies (e.g. twins, triplets)	0.133	**0.570**	0.490	0.653
Provide information and support for women/families who are bereaved (maternal death, stillbirth, pregnancy loss, neonatal death, congenital abnormalities)	-0.086	**0.506**	0.230	0.452
Provide appropriate and timely first-line treatment for any complication detected and refer for further management	0.277	**0.502**	0.592	0.676
Support and educate parents during transport/transfer of newborn or during times of separation	0.323	**0.478**	0.622	0.680
Provide emergency treatment of late post-partum hemorrhage, and refer if necessary	0.260	**0.452**	0.543	0.615
Provide immediate care to the newborn, including cord clamping and cutting, drying, clearing airways, and ensuring that breathing is established	0.327	0.351	0.547	0.556
**Cronbach alpha**	**0.883**	**0.858**		

Bold values indicate major loadings after rotation. Pattern matrix shows the unique contribution of each variable to components. Structure matrix shows the overall correlation of each variable with components. Components are the dimensions derived from the data that explain variance.

The first component with 18 items comprised routine postnatal assessments, information and education as well as initiating breastfeeding. Ten items came from the postnatal section [information and documentation (7), routine maternal check-ups (2), breastfeeding initiation (1)] and eight items from the newborn section [routine newborn care (5), information and documentation (3)].

The second component contained 11 items relating to identifying and acting on postnatal complications and supporting parents with special needs, such as giving birth to twins or triplets. In the second component eight of the 11 items came from the newborn section [identifying and acting on illness or emergency needs of the baby (6), and supporting parents (2)] and the rest of the items were from the postnatal section [identifying complications in mother or baby (2) and supporting bereaved parents (1)].

In the next step, the subscales formed from the components in all the three areas (antenatal, intrapartum and postpartum/newborn care) were analyzed in relation to students’ background characteristics (age, having children, civil status, number of births attended, length of experience as a nurse, medical faculty or not). Only age showed an association with certain components, with younger students (<31 years) reporting higher confidence in ‘Performing routine midwifery assessments during pregnancy’ compared to older students (≥31 years) [mean: 27.43 (SD=3.59) vs mean: 26.01 (SD=3.97), p=0.004]. Similarly, younger students showed higher confidence in the subscale ‘Managing physiological birth and providing support’ [mean: 65.41 (SD=8.25) vs 62.85 (SD=7.89), p=0.01], and finally in the subscale ‘Managing complicated labor and birth’ [mean: 20.37 (SD=5.38) vs mean: 19.05 (SD=4.80), p=0.046]. In the other subscales, there were no statistically significant associations with students’ background characteristics.

## DISCUSSION

The psychometric evaluation of the COMICE questionnaire in the Swedish context confirms its usefulness for measuring midwifery students’ confidence and identifying underlying components within the instrument. The PCA of the COMICE questionnaire identified two components for antenatal and postnatal/newborn care each and three components for intrapartum care. Age of the students was related to some of the subscales in antenatal and intrapartum care.

### Antenatal care

Compared with the international studies that used the COMICE questionnaire, some similarities were found. The wording of subscales/components require fantasy and intuition. The two components in antenatal care were labelled: 1) ‘Performing routine midwifery assessments during pregnancy’, and 2) ‘Identifying fetal and maternal risk factors and educating parents’. In the original study from India^10^ where 633 students participated, three components were identified in antenatal care and labelled: 1) Assess maternal and fetal health, 2) Identify antepartum risks, and 3) Provide counselling and health education to women and families. The last two components are fairly similar to our second component. Midwifery might not be a strong contributor in the Indian study, where the midwifery education is incorporated with the nursing education and only 8% of the sample wanted to work within the field of midwifery after completed education^10^. The results from the study with 1407 participating students from seven African countries, where there are three types of midwifery education also provided components with a semantic similar wording as in the present study^13^. For antenatal care three components were identified in the African study: 1) Identify fetal and maternal risk factors, 2) Educate parents, manage and document emergent complications, and 3) Physical assessment and nutrition^13^.

### Intrapartum care

The three components of intrapartum care identified in the present study were labelled: 1) ‘Managing physiological birth and providing support’, 2) ‘Managing complicated labor and birth’, and 3) ‘Identifying and providing lifesaving measures’. In the Indian as well as the African studies^10,14^, intrapartum care was divided into two parts: the first and second stage of labor, and the third stage of labor. In both studies, the labelling of the components captured the physiological stages as well as the complicated stages, which is quite similar to the components in the present study. In both these studies, there were different types of education, and the result showed that students in direct entry programs and those with post-nursing education were more likely to be highly confident compared to students in integrated nurse-midwifery education^10,14^. There were also some differences between the two studies. Students in the Indian study reported low levels of confidence; diploma students were 2–4 times more likely to have high confidence compared to students at the Bachelor’s level^10^. In the African study, Bachelor’s students and those within direct entry programs were more likely to be confident in intrapartum care^14^.

### Postnatal and newborn care

In the area of postnatal and newborn care, the two components were labelled: 1) ‘Managing uncomplicated postpartum and newborn care and educating parents’ and 2) ‘Managing complicated postpartum and newborn care’. Both the Indian^10^ and the African studies^15^ divided postnatal and newborn care. The wording of the components was quite similar and described basic postnatal care, education of parents and postnatal complications^10,15^. For newborn care, there was one component in the African study^15^ that differed from the Indian^15^ as well as the present study. One component comprised newborn care when the mother was infected with HIV. This component is country-specific due to the high prevalence of HIV in African countries^22^. Those particular items about HIV were omitted in the Swedish questionnaire, as HIV infections are not as common in Sweden.

### Students’ background characteristics

We found no major differences between the students’ background characteristics and the components, with the exception of age, where younger students seemed to be more confidence compared to their older counterparts. In the Indian study, the 633 included students were much younger compared to the Swedish sample, with a median age of 21 years^11^. In the African studies^13-15^, nearly half of the 1407 students were aged 18–25 years. The median age of 31 years in the present study is therefore difficult to compare between the countries. There are indications that younger individuals might be more confident than older individuals in certain contexts^23,24^, but other factors such as the quality of the education or individual learning styles might be more important. However, it has been suggested to the government^25^ that Swedish midwifery education should change into a direct entry program; as such, the country would have a younger midwifery population that could serve society for a longer time, as they would not have to take the detour through the three-year long nursing education.

### Strengths and limitations

This study is compromised by its cross-sectional design and the self-reported nature of the Swedish COMICE questionnaire, which limits the generalizability. Another limitation is the time span since the original study was conducted. The scope of practice and the midwifery education are, however, the same as they were during the data collection. The stepwise procedure used to translate and culturally transform the original COMICE into the Swedish language inspired by Brislin^17^ was a strength, even if it also resulted in the reduction of items that made no sense in a Swedish educational context. We believe that the essence of the COMICE is kept in the Swedish version. The comparison with the studies performed in India and seven African countries showed semantic similarities but are difficult to compare with the Swedish sample. In both India and the African countries, there are a variety of different types of midwifery education, and the length of the programs differs. Midwifery students’ confidence seemed to be higher, at least in the African studies^13-15^, compared to students in the Swedish sample, and it is possible that cultural norms could make students give socially desirable answers as shown^26^. Another strength of the study is the national representativeness, with all midwifery programs participating and the 76% response rate^27^. The Indian and the African studies comprised much larger sample sizes. Nevertheless, the recommended ratio of items to participants was sufficient. Nunnally^28^ recommends a ratio of 1:10, while Tabachnick and Fidell^29^ suggest that 1:5 is enough. Translated into the present study, the number of items ranged from 17–32 in the areas studied, thus they are still within the recommended range. The Cronbach alpha values for the seven identified components ranged from 0.777 to 0.917, suggesting good reliability, as six out of seven components yielded a Cronbach alpha value of more than 0.80, which is regarded as preferable^18^.

## CONCLUSIONS

The widespread use of the COMICE questionnaire shows similarities in the labelling of the components. Although these studies identified different numbers of components, we believe that the semantic coverage captures the main features of antenatal, intrapartum and postpartum/newborn care across all studies. This finding supports the Swedish version of the COMICE questionnaire as a tool to measure and understand confidence among midwifery students in a Swedish context.

## Data Availability

The data supporting this research are available from the authors on reasonable request.
